# Probable Postsurgical Parsonage–Turner Syndrome Following Endoscopic Endonasal Pituitary Surgery: A Diagnostic Challenge Case Report: Illustrative Case

**DOI:** 10.1155/crnm/1964813

**Published:** 2026-08-25

**Authors:** Mousa Javidialsaadi, Alexi Obillo, Alec Germanwala, Andrew S. Ghannad, Ronak H. Jani, Isaac Ng, Vikram C. Prabhu, Chirag R. Patel, Anand V. Germanwala

**Affiliations:** ^1^ Department of Neurological Surgery, Loyola University Medical Center, Maywood, Illinois, USA, loyolamedicine.org; ^2^ Loyola University Chicago Stritch School of Medicine, Maywood, Illinois, USA, luc.edu; ^3^ Department of Otolaryngology, Loyola University Medical Center, Maywood, Illinois, USA, loyolamedicine.org

**Keywords:** brachial neuritis, case report, endoscopic endonasal surgery, Parsonage–Turner syndrome

## Abstract

Brachial neuritis is a rare inflammatory brachial plexopathy characterized by acute and severe shoulder or upper‐extremity pain followed by progressive weakness and sensory disturbances. Commonly associated with viral illness, vaccination, or trauma, it can also occur as a postoperative complication. A 65‐year‐old gentleman presented with visual loss from a large recurrent pituitary adenoma and underwent an uncomplicated resection. Shortly after surgery, he developed swelling and pain in the right forearm and hand, which later evolved into patchy sensory loss and progressive weakness throughout the arm. Examination demonstrated proximal and distal weakness, muscle wasting, and multifocal sensory deficits without signs of cervical radiculopathy, myelopathy, or carpal tunnel syndrome. Pregabalin and gabapentin provided minimal symptom relief. Electromyography and nerve conduction studies performed 6 weeks later showed a moderate right median mononeuropathy and chronic, inactive C7 radiculopathy, findings that did not fully explain his clinical picture. The clinical presentation was most consistent with probable postsurgical Parsonage–Turner syndrome after exclusion of structural and compressive causes, and the patient improved with physical and occupational therapy, achieving near‐complete recovery at 6 months. While early recognition is essential, as electrodiagnostic or radiographic studies may be nondiagnostic, timely therapy can facilitate meaningful functional recovery.

## 1. Introduction

Brachial neuritis, eponymously known as Parsonage–Turner syndrome (PTS), is a rare and often under‐recognized neurological disorder of unclear etiology asymmetrically affecting the brachial plexus. The incidence of PTS is estimated to be approximately 1.64 per 100,000 individuals annually, with a higher prevalence in men, particularly between the third and seventh decades of life; rare cases in children have been reported as well [1]. While the precise etiology remains unclear, brachial neuritis is widely believed to be immune‐mediated. Numerous putative triggers have been associated with its onset, including viral infections (e.g., parvovirus B19, cytomegalovirus, and HIV), vaccination, strenuous physical activity, trauma, and surgical procedures [2–5]. A hereditary form responsible for some cases has been linked to mutations in the SEPT9 gene [6].

Clinically, brachial neuritis is characterized by an acute onset of excruciating shoulder aching and upper arm pain, typically unilateral, followed by muscle weakness, atrophy, and variable sensory deficits in the affected limb [1, 7]. It is usually unilateral, although rare cases of bilateral brachial neuritis have been reported. The upper trunk of the brachial plexus is most involved, resulting in weakness and atrophy of the shoulder girdle and proximal arm muscles such as the deltoid, biceps, supraspinatus, and infraspinatus. Though often self‐limiting, the recovery period is highly variable, ranging from months to several years, and in some cases, patients may not regain their baseline neurological function.

Although postsurgical PTS has been described following a variety of procedures, including orthopedic and spinal operations, its occurrence after endoscopic endonasal skull base surgery remains rarely discussed. This case broadens the surgical contexts in which postoperative immune‐mediated brachial plexopathy should be considered and highlights diagnostic challenges when electrodiagnostic findings are inconclusive [8]. In addition, atypical presentation of brachial neuritis may also occur, and lack of familiarity with this condition can often lead to incorrect diagnosis or ill‐conceived surgical intervention [9]. We present the case of a patient with a recurrent giant pituitary adenoma who underwent an uncomplicated endonasal resection of the tumor but subsequently developed brachial neuritis affecting the right upper extremity. Electrodiagnostic and radiographic studies were not definitive, and the diagnosis was considered most consistent with postsurgical PTS based on clinical presentation after exclusion of structural causes, as reported previously in similar cases [9].

## 2. Case Presentation

**FIGURE 1:**
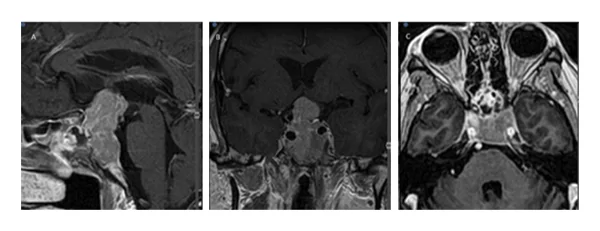
Preoperative MR images: (A–C) preoperative postcontrast T1‐weighted images demonstrate a recurrent pituitary adenoma with clival, sellar, suprasellar, and retrochiasmatic extension and compression of the optic chiasm. Axial (A), coronal (B), and sagittal (C) views show upward displacement of the third ventricle.

This 65‐year‐old right‐handed gentleman underwent a prior microscopic transsellar subtotal resection of a pituitary macroadenoma in 2010 at an outside institution. Following this initial surgery, he had an improvement in his visual field deficits but required long‐term therapy with hydrocortisone and levothyroxine. He presented approximately 14 years later with a gradual deterioration of his peripheral vision. Magnetic resonance imaging studies revealed a large neoplasm in the sella with suprasellar extension and significant chiasmatic compression and invasion of the left cavernous sinus Knosp Grade 3 (Figure 1A–C), and he was referred to our institution. On examination, he was noted to have poor visual acuity with only visible hand motion in the left eye and loss of vision with sparing of the central and inferonasal quadrant vision in the right eye. He was also noted to have gynecomastia with low testosterone, IGF‐1, and T3 levels; the rest of his pituitary hormone studies were within normal limits. He underwent an uneventful endoscopic endonasal resection of the tumor. The pathology was consistent with a pituitary adenoma. Intraoperative neurophysiological monitoring (electroencephalogram, somatosensory evoked potentials, and visual evoked potentials) remained stable throughout the duration of the procedure. Reconstruction of the sella floor was performed with a vascularized nasoseptal flap in an uncomplicated manner.

**FIGURE 2:**
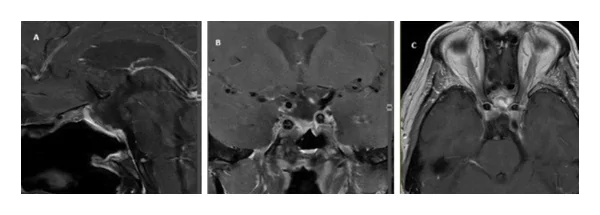
Postoperative MR images: (A–C) postoperative postcontrast T1‐weighted images demonstrate near‐total resection of the pituitary adenoma with decompression of the optic chiasm and floor of the third ventricle. Axial (A), coronal (B), and sagittal (C) views show minimal residual tumor within the left cavernous sinus.

Following surgery, he reported stable visual function. A postoperative MRI demonstrated a nice decompression of the optic chiasm with evidence of removal of the clival, sellar, suprasellar, and retrochiasmatic tumor components (Figure 2A–C). Minimal residual tumor was noted within the left cavernous sinus. No areas of cerebral ischemia or hemorrhage were noted. His postoperative course was marked by diabetes insipidus, for which he was started on desmopressin (DDAVP), and he continued on the preoperative doses of hydrocortisone and levothyroxine. The postoperative serum prolactin normalized, likely reflecting resolution of the preoperative stalk effect. His vision gradually improved over the next few days.

**FIGURE 3:**
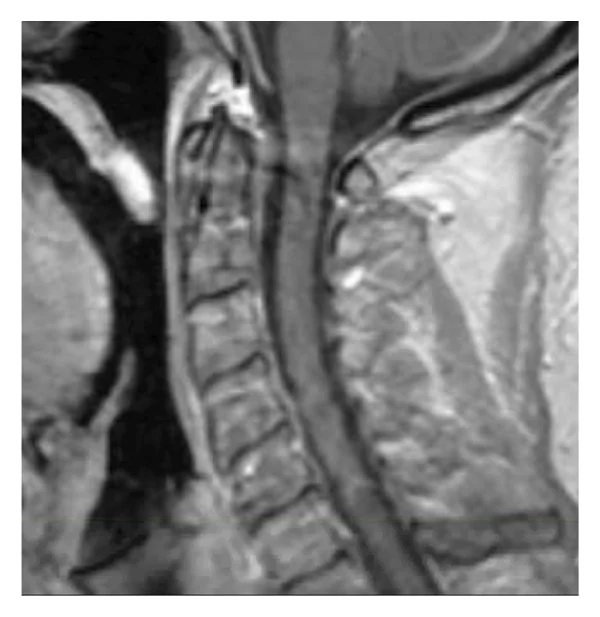
Sagittal T1‐weighted MRI of the cervical spine demonstrating no evidence of significant disc herniation, foraminal narrowing, or cord compression to account for the patient’s right upper extremity deficits.

**FIGURE 4:**
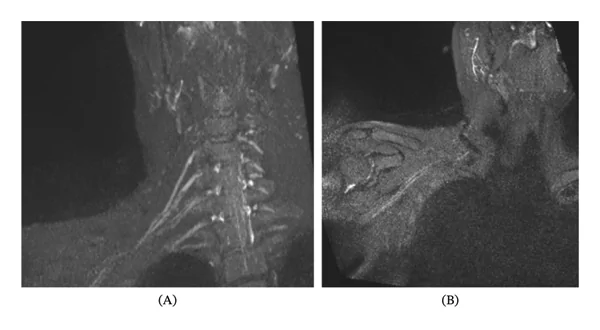
Coronal 3‐D STIR MRI of the right brachial plexus demonstrating no evidence of focal enhancement, dilatation, or hyperintensity within the brachial plexus to explain the patient’s right upper extremity deficits. An unremarkable supraclavicular plexus with trunk formation (A) and infraclavicular plexus with cords leading to terminal branching past the coracoid process in the subpectoral space (B) are depicted.

However, immediately after the procedure, he reported swelling and pain in the right forearm and hand. On postoperative day one, he reported tingling and weakness in digits 1–4 of the right hand with weakness of right‐hand grip strength. He denied having these complaints prior to surgery. He did not report any neck pain or pain radiating down his arm. He has no left upper extremity or lower extremity symptoms or complaints. Based on these symptoms, further imaging was obtained on postoperative Day 2. A cervical spine MRI was obtained to exclude compressive radiculopathy or structural cervical pathology as a cause of the patient’s symptoms. Sagittal T1‐weighted imaging demonstrated no evidence of acute disc herniation, significant foraminal stenosis, spinal cord compression, or nerve root compromise that would explain the diffuse, nondermatomal motor and sensory deficits observed clinically (Figure 3). A brachial plexus MRI did not reveal any abnormalities, including focal enhancement, dilatation, or hyperintensity within the nerves (Figure 4).

Over the next week, the right forearm swelling subsided and was replaced by burning dysesthetic pain in the right hand involving the 2, 3, and 4 fingers. He was started on pregabalin and then transitioned to gabapentin; neither was effective in alleviating the pain completely but did give him some relief. Approximately 6 weeks following onset of symptoms, he was noted to have diffuse (modified Medical Research Council Grade 4‐/5) weakness of the right proximal and distal musculature involving the shoulder girdle, arm, forearm and hand muscles supplied by the suprascapular, axillary, median, ulnar, and radial nerves with patchy sensory loss in the entire right upper extremity. He had no signs of cervical radiculopathy or myelopathy; he did have tenderness over the right cubital and carpal tunnels with a positive Tinel’s sign at these locations.

An electromyography/nerve conduction study (EMG/NCS) approximately 6 weeks following the onset of his symptoms revealed abnormal motor unit potentials in the first dorsal interosseous, triceps brachii, cervical paraspinal muscles, and extensor indicis proprius. The median sensory and motor responses exhibit prolonged distal latencies and reduced sensory amplitude with absent signal conduction beyond the wrist. However, the ulnar and radial nerve studies show normal sensory and motor conduction parameters. The EMG/NCS was reported as demonstrating a focal demyelinating process at the wrist involving the right median nerve in the carpal tunnel with a chronic inactive right C7 radiculopathy. However, his clinical examination was more consistent with brachial plexopathy or brachial neuritis. The overall clinical presentation was considered most consistent with probable postsurgical PTS after exclusion of structural and compressive etiologies, with a possible superimposed right carpal tunnel syndrome.

He was continued on gabapentin, and a structured physical and occupational therapy program focused on the right upper extremity was initiated. Over the course of several weeks, he demonstrated gradual improvement in right upper extremity motor and sensory function, with a gradual decline in dysesthetic pain. At the last follow‐up, 6 months following his initial presentation, he had regained full strength and near‐complete sensory function in the right upper extremity with only minimal residual hypesthesia in the right hand, in his 1–4 digits, but mostly the first 3 digits. The gabapentin was eventually discontinued. He continued to report slow but gradual improvement in his visual acuity, and follow‐up imaging showed no signs of pituitary tumor recurrence or progression of the residual neoplasm within the left cavernous sinus.

The unexpected right‐arm symptoms and the lack of an immediate clear and definitive diagnosis were appropriately concerning to the patient. Understanding of the unanticipated events that can occur after medical care, he was very appreciative of the communication and prompt and thorough workup. He was relieved once he started to have symptomatic improvement that did not necessitate any further surgical intervention. He reported that the medical and physical therapy were easily tolerated. For any further procedures in the future, the patient was encouraged to mention this unexpected postoperative course. While initially unsettling, he was grateful to have had an overall good outcome from the suspected brachial neuritis and brain tumor resection.

## 3. Discussion

Brachial neuritis, or PTS, is a rare peripheral nerve disorder that typically presents with sudden onset of severe shoulder or upper arm pain, followed by weakness, atrophy, and patchy sensory changes in the distribution of the brachial plexus [3, 10]. Though the exact pathogenesis remains unclear, an immune‐mediated mechanism has been proposed, often triggered by antecedent viral infections, vaccinations, or surgical procedures. Recent reports have also linked PTS to SARS‐CoV‐2 infection and COVID‐19 vaccination [3, 5]. In rare instances, brachial neuritis may occur as an autosomal dominant hereditary form linked to chromosome 17q24, known as hereditary neuralgic amyotrophy; these patients may suffer repeated attacks of brachial neuritis precipitated by the same preceding events as noted in the idiopathic nonhereditary form of brachial neuritis [9].

Surgery is now recognized as one of the most common antecedent triggers of PTS, accounting for approximately 30% of cases in contemporary series [11]. Most reported postsurgical cases follow orthopedic, spinal, abdominal, or cardiothoracic procedures. While the pathophysiological mechanism is not fully understood, surgical stress is thought to provoke an immune‐mediated inflammatory response affecting the brachial plexus rather than direct mechanical injury, as described in contemporary postsurgical PTS series.

Nevertheless, distinguishing immune‐mediated plexopathy from positioning‐related, ischemic, or compressive neuropathy remains essential in the postoperative setting. Careful evaluation of surgical positioning, intraoperative events, clinical distribution of deficits, and imaging findings is critical to differentiate these entities. During this surgery, the patient was positioned supine with his arms padded and at his side to ensure no inadvertent compression. The blood pressure cuff was on the left arm, and there were no periods of significant hypotension during the procedure. Additionally, the entire surgery was performed with stable and normal intraoperative neurophysiological monitoring, including somatosensory evoked potentials, that can often intraoperatively identify peripheral neuropathies due to poor positioning during surgery. These circumstances refute mechanical or ischemic etiologies.

The typical clinical course of brachial neuritis starts with an acute, severe, aching shoulder and proximal arm pain that may follow an antecedent event such as infection, vaccination, radiation, or surgery and lasts a few days to weeks [9]. The initial pain is severe and aching in nature and unaltered by neck or arm movements. As the pain abates, it gives way to a profound weakness of the shoulder girdle muscles, such as the supraspinatus and infraspinatus, or serratus anterior, and the proximal arm muscles such as the deltoid or biceps. It is less common to note weakness in the distal muscle groups of the forearm and hand but at times, this may be noted. This is followed by a marked atrophy of the involved muscles and lower motor neuron signs with a hypotonic areflexic paresis and fasciculations in involved muscles; differential involvement of muscles supplied by the same nerve is characteristic of brachial neuritis [9]. Sensory loss is less predictable and can be patchy. At times, single nerve involvement may be noted; for example, a mononeuropathy of the axillary, long thoracic, suprascapular, or even the anterior interosseous nerve may be observed. The affliction in brachial or individual nerve neuritis is usually unilateral, but rare cases of bilateral involvement have been reported; similarly, in rare instances, caudal cranial nerves, the phrenic nerve, or lumbar plexus nerves may be affected by this condition [9]. Other variations may also be noted; children may not present with pain preceding the muscle weakness and wasting phase, and familial cases may afflict both genders and tend to have cranial nerve involvement more commonly than in sporadic cases [9].

The differential diagnosis of brachial neuritis includes cervical radiculopathy or entrapment neuropathies. At times, rotator cuff disease may also mimic this presentation, but a key differentiator is that the shoulder pain is worse with shoulder movements, is worse at night, does not resolve with the development of muscle weakness and atrophy, and generally resolves with a subacromial lidocaine injection [9]. Patients with cervical radiculopathy present with neck pain that radiates down the involved upper extremity in a dermatomal pattern and worsens with neck movements or rotation with extension and axial pressure, as in the Spurling’s sign. Furthermore, the pattern of weakness and hypesthesia follows a dermatomal pattern, and imaging studies such as a cervical MRI demonstrate cervical root compromise by a herniated disc or osteophytic bone spur. Electrophysiological studies are also concordant with this pattern of cervical root involvement. Compressive neuropathies are usually localized to specific regions of the extremity, and electrophysiological nerve conduction velocity studies and EMG are very precise in localizing the site of entrapment.

Cervical spine MRI in our patient demonstrated no structural root compression, significant foraminal stenosis, or cord pathology to explain the patient’s diffuse upper extremity deficits. Although brachial plexus MRI or MR neurography may reveal inflammatory changes, typically described as focal enhancement, hyperintense T2 signal, or focal segmental swelling likened to a “string of beads on a rosary,” in some cases of PTS, imaging findings can be normal. Literature notes that inflammatory changes on imaging typically occur after 48 h and can even take up to 2 weeks to be visualized radiographically [12]. Imaging in the chronic phase may demonstrate atrophy and fatty deposits within the involved musculature [12]. Cases of mild neuritis or imaging early in the disease course may be unremarkable. Therefore, absence of radiographic abnormalities does not exclude an immune‐mediated plexopathy, and its diagnosis remains one based on clinical history and examination.

The key to the diagnosis of brachial neuritis is the characteristic chronology of symptoms and clinical examination findings. Electrophysiological studies show denervation changes in affected muscle groups, but NCS may not be diagnostic. Multiple studies suggest that brachial neuritis, or PTS, is primarily a clinical diagnosis, and that electrodiagnostic testing, while supportive, is not definitive or required [9, 13]. In fact, brachial neuritis may be underdiagnosed due to reliance on “specific diagnostic tools,” underscoring the limitations of ancillary testing and the primacy of clinical suspicion [14]. Typical denervation changes on EMG/NCV studies generally appear only about 3 weeks after symptom onset [15], but in some instances, that also may not be seen, as noted in this case. Collectively, these findings support that a clinically classic presentation of PTS can be appropriately diagnosed even when EMG/NCV results are inconclusive or obtained outside the optimal timing window.

In the patient reported here, the semiology of symptoms and clinical presentation suggested brachial neuritis; the electrophysiological studies did not aid in the diagnosis. There did not appear to be an obvious precipitating event other than the surgical procedure. There was no untoward compression of the brachial plexus as he was positioned supine for the surgery with adequate padding of the extremities and no distortion of the cervical spine. His initial presentation of severe burning pain and weakness in the right upper limb and the subsequent distribution of motor weakness and sensory loss were diffuse and nondermatomal [4]. Although nerve conduction velocity studies revealed a possible right median neuropathy at the right carpal tunnel and chronic inactive right C7 radiculopathy, he did not have objective signs of a cervical root injury or median nerve compromise in the carpal tunnel.

The electrodiagnostic findings of median mononeuropathy and chronic inactive C7 radiculopathy did not adequately account for the widespread clinical deficits observed. Additionally, prior to surgery, the patient had no clinical symptoms or signs of a cervical radiculopathy or median neuropathy. Coalescing this with his overall outcome of near complete resolution of all symptoms at 6 months suggests that these EMG findings were purely incidental. Importantly, EMG abnormalities in PTS may be delayed, patchy, or incomplete. Denervation patterns do not always conform to a single trunk or cord distribution, and early studies may underestimate the extent of inflammatory involvement. Thus, incongruent or partial electrodiagnostic findings do not exclude a clinically consistent diagnosis of postsurgical PTS.

The EMG component of the study revealed denervation changes in radial nerve‐supplied muscle. His pattern of diffuse involvement of multiple myotomes and dermatomes was more suggestive of brachial plexopathy. Although brachial plexopathy was not reported, chronic denervation changes involving the posterior and medial cords, including muscles innervated by C7–T1, were noted. Notably, there was no spontaneous activity, indicating subacute rather than ongoing denervation [10, 15]. It is possible that routine NCS may not detect brachial neuritis; however, abnormal compound muscle action potential (CMAPs) may be noted [6].

The treatment of brachial neuritis is usually conservative and nonsurgical; in fact, it is critical to arrive at the correct diagnosis to avoid unnecessary surgical interventions that will not be of benefit to the patient. Corticosteroids may be used during the initial painful phase but may not impact the overall outcome [9]. Our patient was already on hydrocortisone from the presentation and received stress‐dose steroids perioperatively due to his history of anterior pituitary gland insufficiency; this protocol was sufficient to also treat his suspected brachial plexitis. Other options to control pain include narcotic or non‐narcotic analgesics; there is limited and variable evidence regarding the delayed effectiveness of tricyclic antidepressants and antiepileptic medications for brachial neuritis [9]. Once the painful phase abates, intensive occupational and physician therapy is instituted, and this has the ability to improve the outcome. Recovery in brachial neuritis is typically slow and variable; While some patients regain full function, others are left with some persistent weakness or sensory disturbance; studies suggest approximately 36% of patients have meaningful recovery at 1 year, 75% at 2 years and 89% at 3 years [1, 4, 9]. This is a difficult period for the patient, and regular visits with the physician and counseling are beneficial. Recurrence of brachial neuritis is not common other than in the inherited form [9]. Our patient demonstrated gradual functional improvement with physical and occupational therapy; 6 months from initial presentation, his right upper extremity strength and sensory function had nearly normalized. He also reported continued improvement in his vision, and follow‐up imaging showed no evidence of recurrence of the pituitary tumor or progression of the small remnant within the left cavernous sinus.

### 3.1. Diagnostic Considerations and Differentiation From Compressive or Positioning Injury

The immediate postoperative onset of symptoms raises important diagnostic considerations. Modern series often describe postsurgical PTS developing within 48 h to several weeks after surgery, partly to differentiate immune‐mediated plexopathy from intraoperative stretch or compressive neuropathy. However, immune activation may begin rapidly following surgical stress, and early symptom onset does not exclude PTS.

In this patient, several features argued against isolated positioning or compressive injury. He was positioned supine without shoulder abduction or prolonged traction, and no intraoperative events suggesting brachial plexus stretch were reported. The neurological deficits were diffuse and nondermatomal, involving multiple peripheral nerve territories, including suprascapular, axillary, radial, median, and ulnar distributions, which would be atypical for a focal compressive neuropathy.

### 3.2. Lessons

Postsurgical PTS should be considered in patients with acute upper extremity pain and weakness following surgery, even when the operative field is remote from the brachial plexus. The diagnosis is primarily clinical and requires careful exclusion of positioning‐related or compressive neuropathies. Electrodiagnostic studies and imaging may be inconclusive early in the disease course. Recognition of this entity can prevent unnecessary intervention and guide appropriate rehabilitative management.

## Funding

This research did not receive any specific grant from funding agencies in the public, commercial, or not‐for‐profit sectors.

## Ethics Statement

Institutional Review Board approval was received (LU#217627).

## Consent

Written consent for publication was received from the patient and has been archived with the corresponding author.

## Conflicts of Interest

The authors declare no conflicts of interest.

## Data Availability

Data sharing is not applicable to this article as no datasets were generated or analyzed during the current study.
